# Discovery and Optimization of Selective Inhibitors of Meprin α (Part I)

**DOI:** 10.3390/ph14030203

**Published:** 2021-02-28

**Authors:** Shurong Hou, Juan Diez, Chao Wang, Christoph Becker-Pauly, Gregg B. Fields, Thomas Bannister, Timothy P. Spicer, Louis D. Scampavia, Dmitriy Minond

**Affiliations:** 1 Department of Molecular Medicine, The Scripps Research Molecular Screening Center, Scripps Research, Jupiter, FL 33458, USA; SHou@scripps.edu (S.H.); CWang@scripps.edu (C.W.); tbannist@scripps.edu (T.B.); spicert@scripps.edu (T.P.S.); scampl@scripps.edu (L.D.S.); 2 Rumbaugh-Goodwin Institute for Cancer Research, Nova Southeastern University, 3321 College Avenue, CCR r.605, Fort Lauderdale, FL 33314, USA; mdiezi@yahoo.com; 3 Unit for Degradomics of the Protease Web, Institute of Biochemistry, University of Kiel, Rudolf-Höber-Str.1, 24118 Kiel, Germany; cbeckerpauly@biochem.uni-kiel.de; 4 Department of Chemistry & Biochemistry and I-HEALTH, Florida Atlantic University, 5353 Parkside Drive, Jupiter, FL 33458, USA; fieldsg@fau.edu; 5 Dr. Kiran C. Patel College of Allopathic Medicine, Nova Southeastern University, 3301 College Avenue, Fort Lauderdale, FL 33314, USA

**Keywords:** meprin α, meprin β, zinc metalloproteinase, uHTS

## Abstract

Meprin α and β are zinc-dependent proteinases implicated in multiple diseases including cancers, fibrosis, and Alzheimer’s. However, until recently, only a few inhibitors of either meprin were reported and no inhibitors are in preclinical development. Moreover, inhibitors of other metzincins developed in previous years are not effective in inhibiting meprins suggesting the need for de novo discovery effort. To address the paucity of tractable meprin inhibitors we developed ultrahigh-throughput assays and conducted parallel screening of >650,000 compounds against each meprin. As a result of this effort, we identified five selective meprin α hits belonging to three different chemotypes (triazole-hydroxyacetamides, sulfonamide-hydroxypropanamides, and phenoxy-hydroxyacetamides). These hits demonstrated a nanomolar to micromolar inhibitory activity against meprin α with low cytotoxicity and >30-fold selectivity against meprin β and other related metzincincs. These selective inhibitors of meprin α provide a good starting point for further optimization.

## 1. Introduction

**Figure 1:**
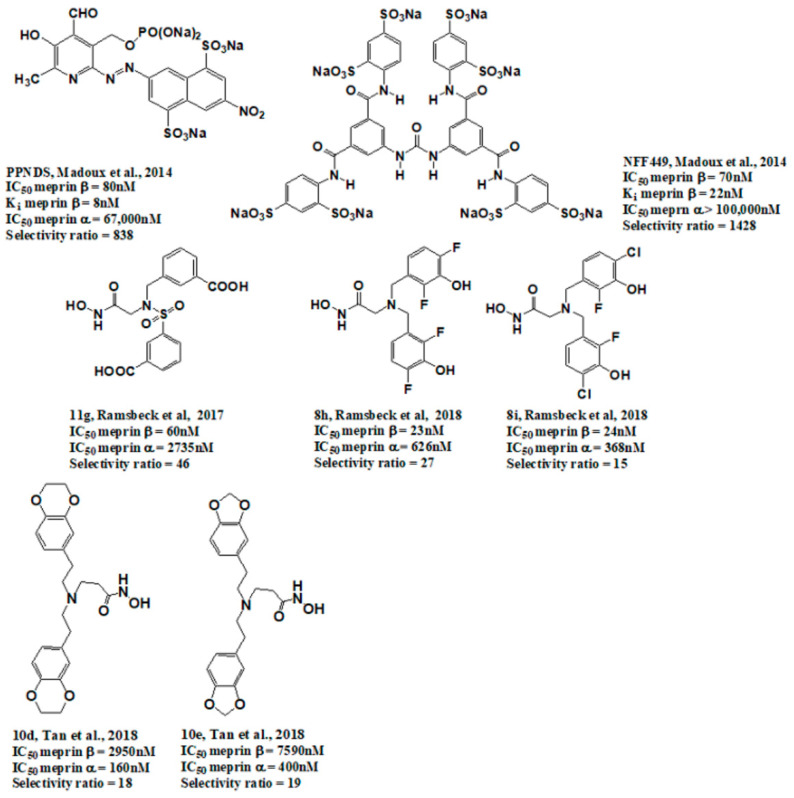
Synthetic selective meprin inhibitors described to date.

Meprin α and meprin β are zinc-dependent proteinases implicated in multiple diseases including cancers [1], fibrosis [2,3], and Alzheimer’s [4,5]. Meprins cleave multiple cytokines and adhesion molecules thus contributing to inflammation and migration of inflammatory cells [6]. Chronic inflammation can lead to the excess deposition of collagen I resulting in fibrosis [7,8]. Meprins have been shown to cleave procollagen I leading to its maturation and deposition in skin and lung [3,9]. The roles of meprins in various processes are mediated via the cleavage of biological molecules. There are examples of common substrates that meprin α and meprin β share amongst themselves [10] and with other proteases [4]. This complicates the understanding of their respective roles in the specific disease scenarios and, as a consequence, their value as targets for drug discovery. To validate either meprin as a target in any particular disease, target modulation by combination of genomic (e.g., knockdown, overexpression) [11] and pharmacologic means (e.g., small molecules) [12] could be useful. However, due to the relatively recent discovery of meprins’ involvement in pathologic conditions there are very few reports of small molecule inhibitor discovery efforts for these enzymes. Kruse et al. [13] reported several known metzincin inhibitors that are capable of inhibiting meprins with some degree of selectivity. However, these inhibitors were not selective for other metzincins, which made their utilization for studying the roles of meprins in various diseases difficult. Our group had reported the first low nanomolar meprin β inhibitors, NFF449 and PPNDS (Figure 1, Ki = 22 nM and 8 nM, respectively), with ~100-fold selectivity against meprin α and good selectivity against adamalysins and matrixins [14]. Ramsbeck et al. (2017) reported the low nanomolar selective meprin β inhibitor, 11 g, with 46-fold selectivity against meprin α (Figure 1, IC50 = 2735 nM and 60 nM for meprin α and β, respectively) with good selectivity against adamalysins and matrixins [15]. They also reported improved compounds based on the same scaffold [16] (Figure 1). The best compounds from this series, 8 h and 8i, are 27-fold and 15-fold selective against meprin α (IC50 = 23 nM and 626 nM for 8 h and 24 nM and 368 nM for 8i, for meprin β and α, respectively). A measure of 200 µM of either inhibitor had only limited effect on MMP and ADAM activity, but IC50 values were not reported. Tan et al. (2018) reported the first selective inhibitors of meprin α, 10d and 10e, with 18- and 19-fold selectivity against meprin β [17] (Figure 1). Herein we report the results of a large-scale parallel high-screening throughput effort to discover novel inhibitors of meprin α and meprin β.

## 2. Results

### 2.1. Assay Miniaturization and Optimization in 1536 Well Plate Format

**Figure 2:**
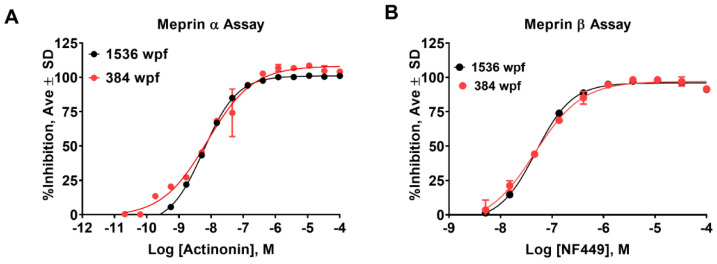
Assay recapitulation in 1536 well plate format. Concentration response studies in 384 and 1536 well plate formats showed similar potency of pharmacological controls for (**A**) meprin α (actinonin) and (**B**) meprin β (NF449) assays. Both assays were performed in triplicate.

The meprin α and meprin β assays, which utilize the substrates (Mca)-YVADAPK-(K-ε-Dnp) and (Mca)-EDEDED-(K-ε-Dnp), respectively, have been described previously [14]. To enable an ultra-high-throughput screening (uHTS) campaign, we proceeded to miniaturize both assays to 1536 well plate format (wpf). First, we recapitulated the assays in 1536 well plate using reagents at the same concentrations as in 384 well plate format assays by scaling the volume down by the factor of 2.5. This resulted in the final volume of the assays of 4 µL. The meprin α assay in 1536 well plates demonstrated a lower signal-to-basal (S/B) ratio than in 384 well plates (1.85 vs. 2.3, respectively), but a better Z’ value (0.76 vs. 0.6, respectively), suggesting that the assay is very suitable for large-scale HTS [18]. Actinonin’s IC50 values were within 2-fold of each other (5.7 nM and 11 nM for 1536 and 384 well plate format, respectively) (Figure 2A and Table 1).

Meprin β assay exhibited greater S/B in 1536 wpf than in 384 wpf (6.9 vs. 4.4, respectively), while Z’ factor values were identical at 0.9. NFF449 IC50 values were 48 nM and 53 nM for 1536 and 384 wpf, respectively (Figure 2B and Table 1). Despite excellent Z’ values in the 1536 wpf in both assays, we wanted to ensure an optimal balance between robustness and sensitivity; in particular with meprin α.

**Figure 3:**
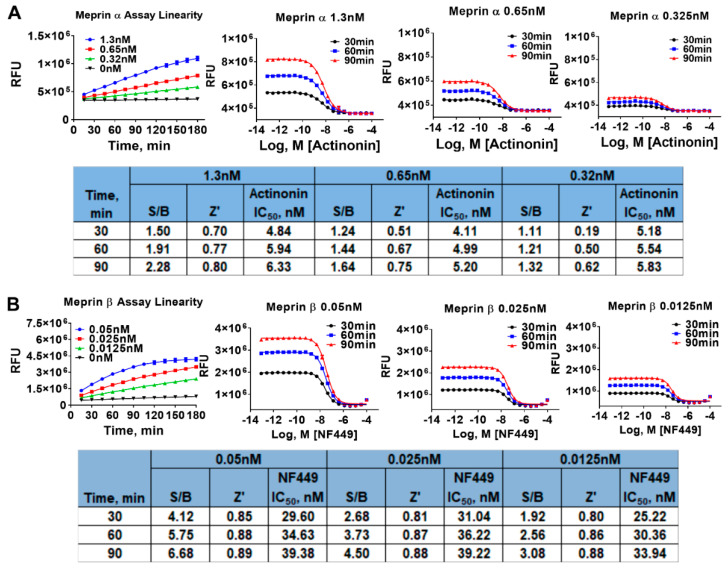
Enzyme concentration and time of reaction optimization experiments in 1536 wpf. (**A**) Meprin α 1536 wpf assay optimization study. (**B**) Meprin β 1536 wpf assay optimization study. Experiments repeated twice, n=4. S/B-signal-to-basal ratio.

First, both assays were run for 180 min at three different enzyme concentrations including the concentrations at which the assays were recapitulated in 1536 wpf (1.3 nM and 0.05 nM for meprin α and meprin β, respectively). QC parameters (Z’ and S/B) and IC50 values of pharmacological controls (actinonin and NFF449) were calculated at 30, 60, and 90 min of the reaction time. The meprin α assay displayed the best S/B values after 90 min of reaction time using 1.3 nM enzyme; however, the reaction progress curve was not linear at the 90 min time point (Figure 3A). This suggested that while longer reaction times and higher than 1.3 nM enzyme concentration may lead to somewhat better S/B values, the assay sensitivity may suffer due to a nonlinear relationship between signal and proteolysis inhibition. Therefore, to ensure optimal assay sensitivity, we chose 60 min reaction end point and 1.3 nM meprin α as final assay conditions for the primary HTS campaign.

The meprin β assay progress curve was hyperbolic rather than linear at 0.05 nM and 0.025 nM enzyme; therefore, we chose 0.0125 nM enzyme concentration where assay linearity was demonstrated (Figure 3B). Z’ and S/B values were acceptable at 60 min reaction end point (0.86 and 2.6, respectively). IC50 values of NFF449 were not significantly affected by the variations of reaction length and meprin β concentrations.

**Figure 4:**
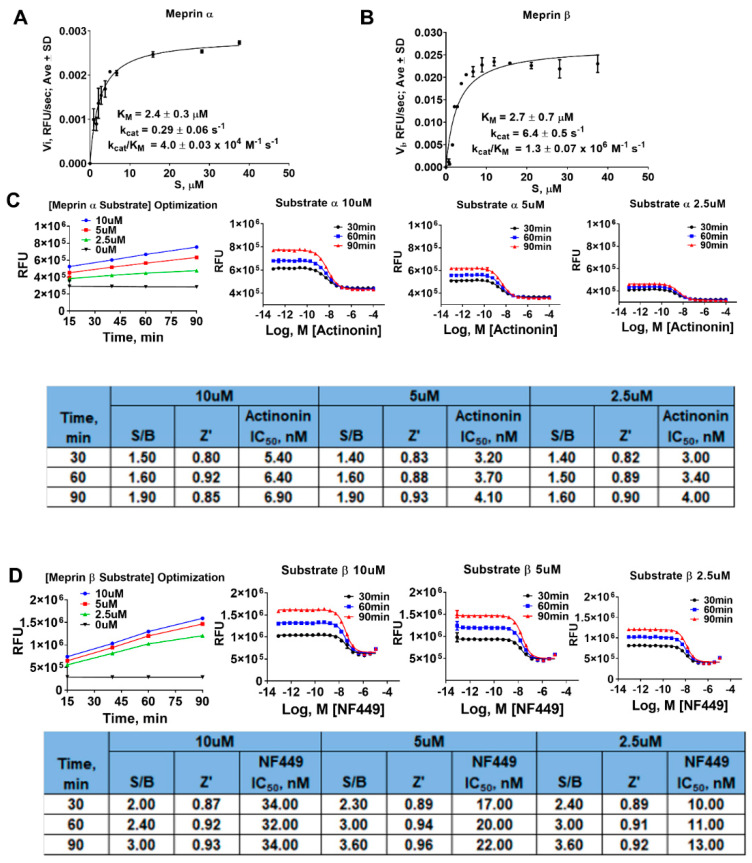
Substrate concentration optimization experiments in 1536 wpf. Results of kinetic studies of (**A**) meprin α and (**B**) meprin β hydrolysis of respective substrates. (**C**) Meprin α 1536 wpf assay optimization study. (**D**) Meprin β 1536 wpf assay optimization study. Experiments repeated twice, *n* = 4.

Next, we performed substrate optimization to achieve balanced assay conditions defined as [S]/KM = 1 [19]. In order to do that, we first determined kinetic parameters of proteolysis of meprin α and meprin β substrates by the respective enzymes (Figure 4A,B). Meprin α and meprin β proteolysis exhibited similar KM values (2.4 ± 0.3 µM and 2.7 ± 0.7 µM, respectively) suggesting the need for optimization of both assays’ substrate concentration. Meprin β exhibited >20-fold faster turnover of its substrate than meprin α (6.4 ± 0.06 s^-1^ versus 0.29 ± 0.06 s-1, respectively) which is consistent with >100-fold difference in enzyme concentrations for meprin α and meprin β assays (1.3 nM versus 0.0125 nM, respectively). To optimize substrate concentrations, both assays were run for 90 min at three different substrate concentrations (10, 5, and 2.5 µM) which included the concentration at which the assays were recapitulated in 1536 wpf (10 µM for both meprin α and meprin β) and the concentration approximating [S]/KM = 1 condition (2.5 µM). Enzyme concentrations were fixed at 1.3 nM for meprin α and 0.0125 nM for meprin β. QC parameters (Z’ and S/B) and IC50 values of pharmacological controls (actinonin and NFF449) were calculated at 40, 60, and 90 min of the reaction time (Figure 4C,D). The 2.5 µM substrate condition resulted in increased apparent potency of pharmacological controls for both assays (2-fold for actinonin in the meprin α assay and 3-fold for NFF449 in the meprin β assay). This suggested that 2.5 µM substrate concentrations result in greater assay sensitivity. Assay QC parameters (S/B and Z’) at 2.5 µM substrate concentrations did not differ significantly from assays run at 10 µM substrate concentrations; therefore, we chose 2.5 µM substrate concentrations as a final assay condition.

### 2.2. Online Robotic Pilot Study

To ascertain the readiness of the assays for a large-scale screening effort, a small pilot screen was conducted using Kalypsys GNF integrated online robotic platform (San Diego, CA, USA) [20]. Overall, ~39,000 compounds were tested using 31 assay plates in both meprin α and meprin β assays. Both assays performed well on the Kalypsys robotic system, as the meprin α assay average Z’ and S/B were 0.88 ± 0.03 and 2.9 ± 0.07, respectively, while the meprin β assay average Z’ and S/B were 0.91 ± 0.03 and 4.5 ± 0.17, respectively. The number of hits identified in the meprin α and meprin β assays were 169 and 260, respectively, which constituted 0.43% and 0.67% hit rates, respectively. After removal of duplicates, Venn analysis showed that 37 compounds inhibited both meprins, while there were 129 compounds selectively inhibiting meprin α and 220 compounds selectively inhibiting meprin β, suggesting that selective probes for both enzymes could be discovered. This also suggested that both assays were ready for large scale effort.

### 2.3. Primary HTS Campaign

**Figure 5:**
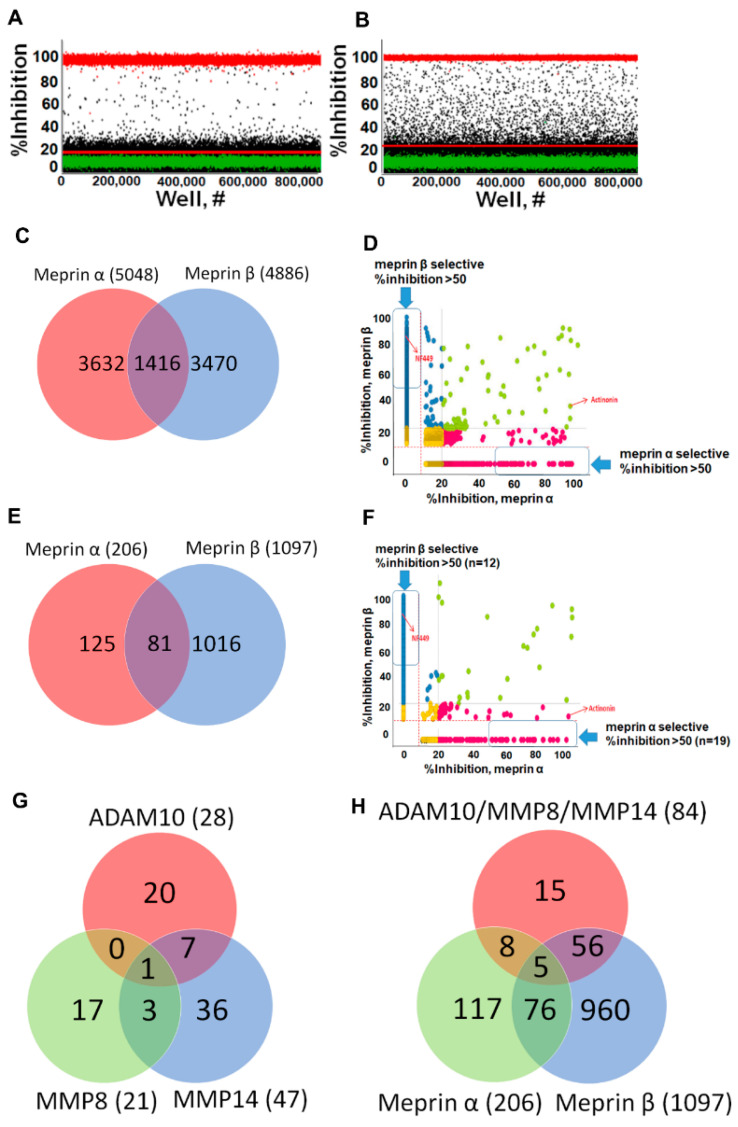
Primary uHTS campaigns. Scatter plots of (**A**) meprin α and (**B**) meprin β primary campaigns. Overall, >650,000 compounds were screened in singlicate against each target. (**C**) Venn diagram of meprin α and meprin β uHTS hits shows 1416 nonselective hits, 3632 meprin α and 3470 meprin β nominally selective hits. (**D**) Correlation plot of meprin α and meprin β actives demonstrates distribution of hits. (**E**) Venn diagram of meprin α and meprin β confirmation assays shows confirmed 81 confirmed nonselective hits, 125 meprin α and 1016 meprin β confirmed selective hits. Each confirmation assay was performed in triplicate. (**F**) Correlation plot of meprin α and meprin β actives demonstrates distribution of hits. (**G**) Venn diagram of meprin α and β hits screen against the counter targets MMP-8, MMP-14, and ADAM10. The 84 meprin actives inhibited one of three counter targets. (**H**) Venn diagram of meprin α and β hits versus three combined counter targets. A total of 117 compounds selectively inhibited meprin α while 960 compounds selectively inhibited meprin β.

Primary HTS campaigns were conducted using The Scripps Research Institute proprietary library of 649,570 compounds using both meprin α and meprin β assays [21]. Overall, 522 plates were used for each assay with excellent QC parameters (average Z’ = 0.86 ± 0.04 and average S/B = 2.8 ± 0.09 for meprin α assay and average Z’ = 0.88 ± 0.03 and average S/B = 4.4 ± 0.27 for meprin β assay). IC50 values of control compounds were reproducible with literature and our preliminary experiments (meprin α actinonin IC50 = 2.9 ± 0.12 nM, *n* = 11 plates; meprin β NF449 IC50 = 10.4 ± 0.85 nM, *n* = 11 plates). Using hit cutoffs derived from the average and 3 standard deviations of the activity of all samples tested which were 10.76% and 14.33% for the meprin α and meprin β assays, 5064 and 4929 hits were identified which constituted hit rates of 0.78% and 0.76%, respectively. It was noted that the majority of meprin α hits exhibited a percentage inhibition close to the hit cutoff, whereas meprin β hits were distributed evenly in the range of 20−100% inhibition (Figure 5A,B).

After removal of a handful of duplicates, Venn analysis showed that 1416 compounds inhibited both meprins, while there were 3632 compounds selectively inhibiting meprin α and 3470 compounds selectively inhibiting meprin β (Figure 5C). Correlational analysis showed 48 and 39 compounds selectively inhibiting meprin α and meprin β, respectively, with a percentage inhibition ≥ 50 (Figure 5D).

### 2.4. Hit Confirmation and Prioritization

For the confirmation assays all compounds that inhibited either of the meprins with >20% inhibition were selected. Confirmation assays were done at a single concentration point in triplicate. Out of 2378 total compounds tested in confirmation assays, only 206 confirmed activity against meprin α and 1097 confirmed activity against meprin β constituting 8.7% and 46.1% confirmation rate for meprin α and meprin β, respectively. The low confirmation rate for meprin α was not unexpected due to the majority of meprin α hits from the primary campaign being close to the hit cutoff (Figure 5A).

Venn analysis showed that 81 compounds inhibited both meprins, while there were 125 compounds selectively inhibiting meprin α and 1016 compounds selectively inhibiting meprin β (Figure 5E). Correlational analysis showed 19 and 12 compounds selectively inhibiting meprin α and β, respectively, with ≥50% inhibition (Figure 5F). Overall, 827 compounds exhibited >20% inhibition.

It was also noted that the majority of the most active hits for each enzyme were potential Zn-binders due to the presence of hydroxamate and reverse hydroxamate moieties. Compounds acting via Zn binding may be undesirable due to clinical trial failures observed previously based on a lack of selectivity, toxicity, and metabolic instability. To prioritize selectivity, we introduced additional assays to help with triaging the compounds to ascertain that we are not biasing for nonselective compounds. We utilized ADAM10, MMP-8, and MMP-14 as the most relevant counter targets. The counter screens were conducted in triplicate using the same 2378 compounds that were tested in confirmation assays.

Venn analysis showed that 84 meprin actives inhibited at least one counter target (Figure 5G), while there were 117 compounds selectively inhibiting meprin α and 960 compounds selectively inhibiting meprin β (Figure 5H) and 14 and 75 compounds selectively inhibiting meprin α and meprin β, respectively, with ≥50% inhibition. Cheminformatics analysis of the Scripps HTS assay database containing hundreds of biological assay results showed that 660 out of 1237 confirmed hits were not promiscuous; meaning they hit in less than 5 other assays. Out of these 660 compounds 536 were meprin α active and 195 were meprin β active. Medicinal chemistry triage suggested that 289 compounds out of 536 meprin α actives were tractable, while out of 195 meprin β actives 180 were tractable, which constitutes 469 total tractable compounds. Removal of 62 duplicates left us with 407 unique compounds of which 404 were available for concentration response studies. Despite the majority of top actives from the 2378 primary HTS hits being potential Zn-binders, the hit rate in counter screens was < 2.0% (Figure 5G,H) suggesting low metzincin promiscuity of meprin hits.

We conducted concentration response studies of 404 compounds in meprin α and β assays using 10-point 3:1 serial dilutions starting at the highest concentration of 17.4 µM in triplicate. Out of 404 tested compounds, 13 exhibited IC50 values < 1 µM and 47 < 5 µM in in both meprin α and meprin β assays.

To pick compounds for further characterization and probe development we used a cutoff of IC50 values < 10 µM against either meprin and 10-fold selectivity window for meprin α or meprin β. Additionally, we picked the top selective compounds with IC50 values < 10 µM that had no apparent Zn-binding moieties. More specifically, we prioritized selective compounds without apparent Zn-binding groups (hydroxamates, carboxylates, etc.). Using these criteria, we selected 46 compounds. Interestingly, the majority (42) were selective for meprin β and only 4 were selective for meprin α. These 46 potentially non-Zn-binding compounds were clustered in [21] distinct scaffolds. The most populated scaffold had 9 members suggesting its amenability to medicinal chemistry.

The second group of compounds was chosen based on selectivity between main target (either meprin α or β) and four other tested metzincins (either meprin α or meprin β, ADAM10, MMP-8, and MMP-14) and potency towards the main target (either meprin α or meprin β) regardless of the presence of Zn binders. These criteria yielded 41 compounds belonging to 17 distinct clusters. Interestingly, the majority (32) were selective for meprin α and only 9 were selective for meprin β, which is the opposite trend from non-Zn-binders.

### 2.5. Hit Potency, Selectivity, and Cytotoxicity

We were able to procure 64 out of 87 selected compounds from commercial sources, which we tested in triplicate, 10-point, 3:1 serial dilution concentration response format starting at the highest concentration of 17.4 µM against both meprin α and meprin β. In addition to meprins, we also tested 64 hits against related metzincins (MMP-2, MMP-3, MMP-8, MMP-9, MMP-10, MMP-14, ADAM10, and ADAM17) to ascertain general nonpromiscuity against zinc-dependent proteases.

**Figure 6:**
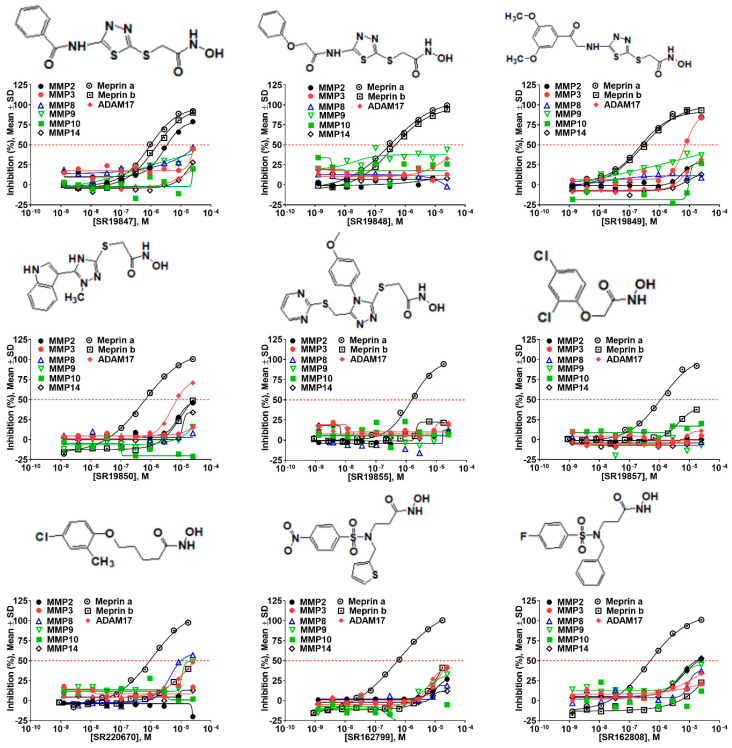
Results of concentration response studies of top potent and selective meprin α inhibitors.

The top nine compounds exhibited IC50 values ≤ 1 µM against meprin α (Table 2). Examination of the structures of meprin α top hits revealed that they fall into four groups (Figure 6), thiadiazole-hydroxyacetamides (SR19849, SR19848, SR19847), triazole-hydroxyacetamides (SR19850, SR19855), sulfonamide-hydroxypropanamides (SR162808, SR162799), and phenoxy-hydroxyacetamides (SR1220670, SR1596857). SR162808 was the most potent and selective inhibitor of meprin α with an IC50 value of 0.446 µM and >30-fold selectivity against meprin β and other tested metzincins (Table 2). Both sulfonamide-hydroxypropanamides (SR162808 and SR162799) exhibited sub-micromolar IC50 values for meprin α inhibition and >30-fold selectivity against meprin β and other tested metzincins.

**Figure 7:**
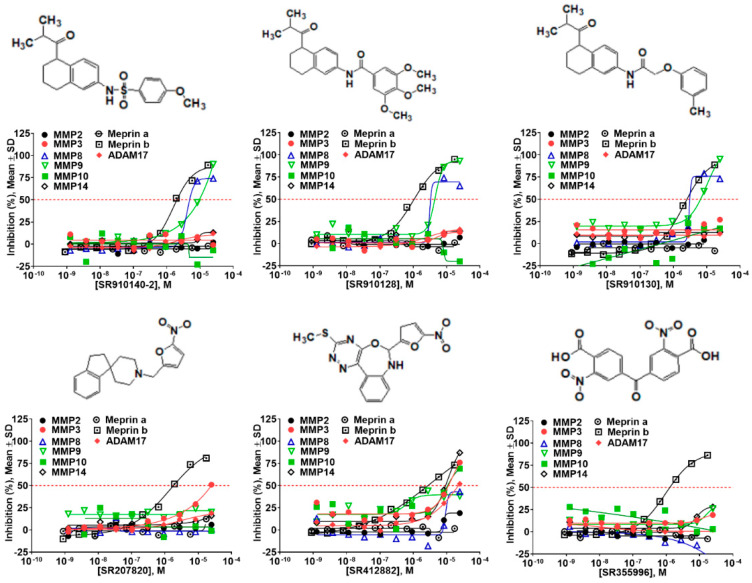
Results of concentration response studies of top potent and selective meprin β inhibitors.

The top meprin β inhibitors belonged to two structural families (Figure 7 and Table 3), isobutyryl-tetrahydronaphthalen-amides (SR910128, SR910130, and SR910140) and nitrofuran-containing compounds (SR207820 and SR412882). Compound SR355996 was the only representative of the bis-nitrobenzoic acid scaffold.

**Figure 8:**
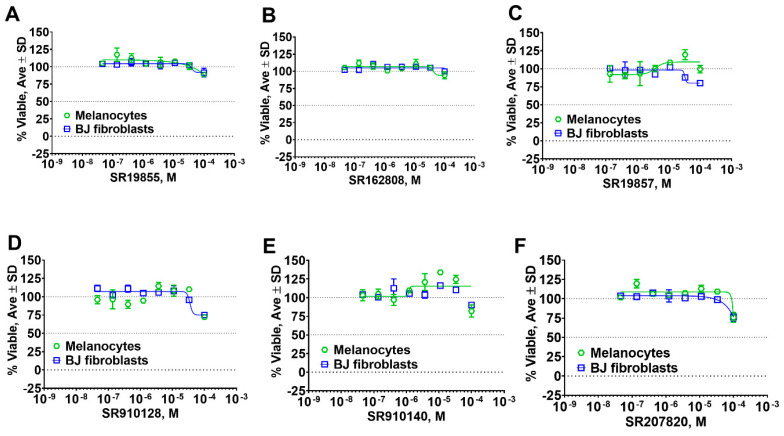
Results of cytotoxicity studies of representative meprin α and meprin β inhibitors. (**A**–**C**) Meprin α inhibitors. (**D**–**F**) Meprin β inhibitors.

We also tested representative compounds from each scaffold for effects on skin fibroblast and melanocyte viability to ascertain cytotoxicity towards various skin cell types. Overall, hits showed either no or very little effect on cell viability (Figure 8) suggesting a lack of general cytotoxicity and amenability of hit chemotypes for the development into in vitro probe for biological studies.

## 3. Discussion

As the result of the uHTS effort we discovered and characterized several novel scaffolds with activity against meprin α and meprin β. All top selective meprin α HTS hits contain a hydroxamate moiety, whereas meprin β hits lack one. Based on the presence of the hydroxamate moiety in meprin α inhibitors it is likely that they act via binding of the active site zinc atom as was demonstrated for numerous other metzincins. Tan et al. [17] proposed the interaction model whereby the hydroxamic moiety of an analog of compounds 10d and 10e (Figure 1) binds zinc and carboxylate moieties interact with residues of the S1 and S1′ subsites. Based on this model, the selectivity of 10d and 10e is derived from differences between meprin α and meprin β S1 and S1′ subsites. Both 10d and 10e structures are symmetric with a central hydroxamate moiety connected via propyl linkers to either terminal benzodioxanes or benzodioxols. Our HTS hits are unlikely to interact with both subsites as the hydroxamate is terminal in all cases. Similar to 10d and 10e, most of the hits (Table 2) have at least one other electronegative moiety in addition to the hydroxamate that could be interacting with positively charged residues in either subsite of meprin α. However, only five (SR19855, SR1596857, SR220670, SR162799, and SR162808) out of nine hits show selectivity for meprin α suggesting that additional interactions may be responsible for selectivity against meprin β.

The most selective and potent meprin α HTS hit, SR162808, exhibited more than 30-fold selectivity against meprin β and other metzincins (Table 2) and no cytotoxicity (Figure 8B). For comparison, 10d and 10e exhibit 18-fold and 19-fold selectivity, respectively (Table 4). Unfortunately, nothing has been reported about their effects on cell viability. For in vivo probe or drug lead development significant selectivity and toxicity windows are extremely important; therefore, SR162808 represents a good starting point for a medicinal chemistry optimization effort.

The HTS-based approach to metalloproteinase or any other inhibitor discovery has inherent limitations and strengths. The strength of HTS is in its ability to assess multiple chemical scaffolds for activity against the target of choice and the ability to select the most promising scaffolds for further optimization. In the event that the chosen scaffold is not amenable to optimization, the researchers can go back to the HTS campaign results and choose additional scaffolds. The main limitation of the HTS approach is that it relies heavily on the composition of the HTS library that is being screened. In our case, we found several novel scaffolds active and selective for both meprins, however, most of these scaffolds contain zinc-binding moieties such as hydroxamates. While hydroxamates have good binding affinity to zinc of the active site, they have their limitations (reviewed in [22]). More specifically, the zinc-binding property of hydroxamates can lead to off-target interactions with other members of metzincin superfamily (e.g., adamalysins, matrixins), unfavorable pharmacokinetic properties, dose-limiting toxicity and metabolic instability, to name a few. However, despite these limitations, there are multiple examples of hydroxamates being used in the clinic (reviewed in [23]). Examples include, but are not limited to histone deacetylase inhibitors (HDACI) panobinostat and bellinostat, deferoxamine, a chelating agent used to treat iron or aluminum toxicity [24].

In conclusion, an HTS campaign led to the discovery of [5] selective meprin α hits belonging to three different chemotypes: triazole-hydroxyacetamides (SR19855), sulfonamide-hydroxypropanamides (SR162808 and SR162799), and phenoxy-hydroxyacetamides (SR1220670 and SR1596857). The chemical diversity of the HTS hits, a good metzincin selectivity profile, and low cytotoxicity suggest that these hits can be developed into more potent compounds for in vivo studies. Further medicinal chemistry optimization and in vivo studies will help determine the value of these scaffolds as probes or leads for drug development. The main value of current HTS hits is in their good selectivity for main target meprin α against a counter target meprin β and related metzincins. This property will help differentiate the roles of these two enzymes in biological and pathobiological processes.

## 4. Materials and Methods

### 4.1. Reagents

MMP-1, MMP-2, MMP-8, MMP-9, MMP-10, MMP-13, MMP-14, ADAM10, ADAM17 and Mca-KPLGL-Dpa-AR-NH2 fluorogenic peptide substrates were purchased from R&D Systems (cat # 901-MP, 902-MP, 908-MP, 911-MP, 910-MP, 511-MM, 918-MP, 936-AD, 930-ADB, and ES010, respectively). All common chemicals were purchased from Sigma. NFF449 was purchased from Tocris (cat# 1391) and actinonin was from Sigma-Aldrich (cat# 01809).

### 4.2. HTS Substrate Synthesis

Meprin α and meprin β substrates ((Mca)-YVADAPK-(K-ε-Dnp) and (Mca)-EDEDED-(K-ε-Dnp), respectively) [25] were synthesized utilizing Fmoc solid-phase methodology on a peptide synthesizer. All peptides were synthesized as C-terminal amides to prevent diketopiperazine formation [26]. Cleavage and side-chain deprotection of peptide-resins was for at least 2 h using thioanisole-water-TFA (5:5:90). The substrates were purified and characterized by preparative RP HPLC and characterized by MALDI-TOF MS and analytical RP HPLC.

### 4.3. Meprins Expression Protocol

Recombinant human meprin α and meprin β were expressed using the Bac-to-Bac expression system (Gibco Life Technologies, Paisley, UK) as described [27,28,29]. Media and supplements were obtained from Gibco Life Technologies. Recombinant Baculoviruses were amplified in adherently growing Spodoptera frugiperda (Sf)9 insect cells at 27 °C in Grace’s insect medium supplemented with 10% fetal bovine serum, 50 units/mL penicillin, and 50 μg/mL streptomycin. Protein expression was performed in 500 mL suspension cultures of BTI-TN-5B1-4 insect cells growing in Express Five SFM supplemented with 4 mM glutamine, 50 units/mL penicillin, and 50 μg/mL streptomycin in Fernbach flasks using a Multitron orbital shaker (INFORS AG, Bottmingen, Switzerland). Cells were infected at a density of 2 × ^106^ cells/mL with an amplified viral stock at a MOI of ~10. Protein expression was stopped after 72 h, and recombinant meprins were further purified from the media by ammonium sulfate precipitation (60% saturation) and affinity chromatography (Streptactin for Strep-tagged meprin α and Ni-NTA for His-tagged meprin β). Meprins were activated by trypsin, which was removed afterwards by affinity chromatography using a column containing immobilized chicken ovomucoid, a trypsin inhibitor.

### 4.4. Meprin α and Meprin β Assays in 384 Well Plate

Both assays followed the same general protocol [14]. A 5 µL measure of 2 × enzyme solution (2.6 and 0.1 nM for meprin α and meprin β, respectively) in assay buffer (50 mM Hepes, 0.01% Brij-35, pH 7.5) was added to solid bottom black 384 low volume plates (Nunc, cat# 264705). Next, 75 nL of test compounds or pharmacological control (actinonin or NFF449) were added to corresponding wells using a 384-pin tool device (V&P Scientific, San Diego, CA, USA). After 30 min incubation at RT, the reactions were started by addition of 5 µL of 2 × solutions of substrates (20 µM, mepin α substrate Mca-YVADAPK-K(Dnp) or meprin β substrate Mca-EDEDED-K(Dnp)). Reactions were incubated at RT for 1 h, after which the fluorescence was measured using the Synergy H4 multimode microplate reader (Biotek Instruments) (λexcitation = 324 nm, λemission = 390 nm).

Three parameters were calculated on a per-plate basis: (a) the signal-to-background ratio (S/B); (b) the coefficient for variation (CV; CV = (standard deviation/mean) × 100)) for all compound test wells; and (c) the Z- or Z’-factor [18]. Z takes into account the effect of test compounds on the assay window, while Z’ is based on controls.

### 4.5. Determination of Kinetic Parameters of Meprin α and Meprin β Mediated Proteolysis of Their Respective Substrates

Substrate stock solutions were prepared at various concentrations in HTS assay buffer (50 mM Hepes, 0.01% Brij-35, pH 7.5). Assays were conducted by incubating a range of substrate concentrations (2–50 µM) with various meprin concentrations at 25 °C. Fluorescence was measured on a multimode microplate reader Synergy H1 (Biotek Instruments, Winooski, VT, USA) using λexcitation = 324 nm and λemission = 393 nm. Rates of hydrolysis were obtained from plots of fluorescence versus time, using data points from only the linear portion of the hydrolysis curve. The slope from these plots was divided by the fluorescence change corresponding to complete hydrolysis and then multiplied by the substrate concentration to obtain rates of hydrolysis in units of μM/s. Kinetic parameters were calculated by nonlinear regression analysis using the GraphPad Prism 8.0 suite of programs.

### 4.6. Meprin α and Meprin β Assays in 1536 Well Plate Format

Both assays followed the same general protocol. A 2 µL portion of 2 × enzyme solution (1.3 and 0.0125 nM for meprin α and meprin β, respectively) in assay buffer (50 mM Hepes, 0.01% Brij-35, pH 7.5) was added to solid bottom black 1536 low volume plates (Corning cat# 7261). Next, 30 nL of test compounds or pharmacological control (actinonin or NFF449) was added to corresponding wells using a 1536 pin tool device (V&P Scientific, San Diego). After 30 min incubation at RT, the reactions were started by addition of 2 µL of 2x solutions of substrates (20 µM, meprin α substrate Mca-YVADAPK-K(Dnp) or meprin β substrate Mca-EDEDED-K(Dnp)). Reactions were incubated at RT for 1 h, after which the fluorescence was measured using the Viewlux multimode microplate reader (Perkin Elmer) (λexcitation = 324 nm, λemission = 390 nm).

Three parameters were calculated on a per-plate basis: (a) the signal-to-background ratio (S/B); (b) the coefficient for variation (CV; CV = (standard deviation/mean) × 100)) for all compound test wells; and (c) the Z- or Z’-factor [18]. Z takes into account the effect of test compounds on the assay window, while Z’ is based on controls.

### 4.7. uHTS Campaign

The miniaturized 1536-well plate format meprin α and meprin β assays were used to screen a collection of approximately 650,000 compounds (The Scripps Research library, La Jolla, CA, USA) on the automated Kalypsys/GNF platform at The Scripps Research Molecular Screening Center (SRMSC, Jupiter, FL, USA: http://hts.florida.scripps.edu/). Both uHTS campaigns were run separately but in a similar manner. Briefly, the first step was the primary screen of all test compounds as singlicates against the meprin α and meprin β target at a final concentration of 7.0 μM. Next, compounds selected as primary hits were cherry-picked and retested in triplicate against the primary screen target and its antitarget (meprin α for the meprin β screening effort, and vice versa) at a same final concentration of 7.0 μM. The additional counter screen assays against related metzincins (MMP-8, MMP-14, and ADAM10) were performed in triplicate at a final concentration of 7.0 μM. The final step was the titration of selected hits as 10-point, 1:3 serial dilutions in both the target and antitarget assay, starting at a final nominal concentration of 17 μM. For all the aforementioned assays, actinonin and NFF449, for meprin α and meprin β, respectively, at a final concentration of 1 µM, were used as a positive control and reference for 100% inhibition. Wells treated with DMSO only were used as negative controls and 0% inhibition reference. The percentage inhibition of each well was then normalized as follows:

%_Inhibition = (RFU_Test_Compound—MedianRFU_Low_Control)/(MedianRFU_High_Control—MedianRFU_Low_Control) * 100(1)

where “Test_Compound” refers to wells containing test compound, “High_Control” is defined as wells treated with either actinonin or NFF449 (*n* = 24) and “Low_Control” as wells containing DMSO only (*n* = 24). All data generated during this effort were uploaded to the SRMSC’s institutional screening database (Assay Explorer, Symyx). Sample to background (S/B) ratios, as well as Z and Z’ values, were calculated on a per plate basis as described 14. Curve fitting and resulting IC50 determinations were performed as previously reported [30].

### 4.8. ADAM10 and ADAM17 Assays

Both assays followed the same general protocol. A 2.5 µL portion of 2 × enzyme solution (20 nM) in assay buffer (10 mM HEPES, 0.001% Brij-35, pH 7.5) was added to solid bottom black 1536 plates (Greiner, cat# 789075). Next, test compounds and pharmacological controls were added to corresponding wells using a 1536 pin tool device (V&P Scientific, San Diego, CA, USA). After 30 min incubation at RT, the reactions were started by addition of 2.5 µL of 2x solutions of substrate (R&D Systems cat#: ES010, Mca-KPLGL-Dpa-AR-NH2, 20 µM). Reactions were incubated at RT for 2 h, after which the fluorescence was measured using a PerkinElmer Viewlux multimode microplate imager (λexcitation = 324 nm, λemission = 390 nm). Final concentration of test compounds in assays was 7.0 µM.

### 4.9. MMP Assays

All assays followed the same general protocol. A 5 µL portion of 2 × enzyme solution (5 nM) in assay buffer (50 mM Tricine, 50 mM NaCl, 10 mM CaCl2, 0.05% Brij-35, pH 7.5) was added to solid bottom black 384 plates (Nunc, cat# 264705). Next, test compounds and pharmacological controls were added to corresponding wells using a 384-pin tool device (V&P Scientific, San Diego, CA, USA). After 30 min incubation at RT, the reactions were started by addition of 5 µL of 2x solutions of substrate (R&D Systems cat#: ES010, Mca-KPLGL-Dpa-AR-NH2, 20 µM). Reactions were incubated at RT for 1 h, after which the fluorescence was measured using the Synergy H4 multimode microplate reader (Biotek Instruments) (λexcitation = 324 nm, λemission = 390 nm).

### 4.10. Cell Toxicity Studies

Test compounds were solubilized in 100% DMSO and added to polypropylene 384 well plates (Greiner cat# 781280). The 1250 of BJ skin fibroblasts (ATCC CRL-2522) and primary melanocytes (ATCC PCS-200-013) were plated in 384 well plates in 8 µL of serum-free media (HybriCare for BT474, EMEM for HEK293). Test compounds and pharmacological assay control (lapatinib) were prepared as 10-point, 1:3 serial dilutions starting at 10 mM, then added to the cells using the pin tool mounted on the Integra 384. Plates were incubated for 72 h at 37 °C, 5% CO_2_ and 95% relative humidity. After incubation, 8 µL of CellTiter-Glo (Promega cat# G7570) was added to each well and incubated for 15 min at room temperature. Luminescence was recorded using a Biotek Synergy H1 multimode microplate reader. Viability was expressed as a percentage relative to wells containing media only (0%) and wells containing cells treated with DMSO only (100%). Three parameters were calculated on a per plate basis: (a) the signal-to-background ratio (S/B); (b) the coefficient for variation (CV; CV = (standard deviation/mean) × 100)) for all compound test wells; and (c) the Z’-factor. IC50 values were calculated by fitting normalized data to sigmoidal log versus response equation utilizing nonlinear regression analysis from GraphPad Prizm 8.

## Figures and Tables

**Table 1:** Comparison of meprin α and meprin β assay parameters in 384 and 1536 well plate formats.

Assay	S/B	Z’	Actinonin IC_50_, nM	NFF449 IC_50_, nM
Meprin α 384 wpf	2.3	0.6	11	>100,000
Meprin α 1536 wpf	1.85	0.76	5.7	>100,000
Meprin β 384 wpf	4.4	0.9	22,000	53
Meprin β 1536 wpf	6.9	0.91	9750	48

**Table 2:** Selectivity testing of meprin α top HTS hits. All units are IC_50_, µM.

Compound ID	Structure	Meprin α	Meprin β	MMP2	MMP3	MMP8	MMP9	MMP10	MMP14	ADAM17
19847	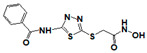	0.892	1.43	2.87	>17	>17	>17	>17	>17	>17
19848	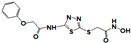	0.335	0.385	>17	>17	>17	>17	>17	>17	>17
19849	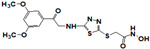	0.218	0.287	>17	>17	>17	>17	>17	>17	8.01
19850	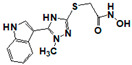	0.564	17	17	>17	>17	>17	>17	>17	5.03
19855	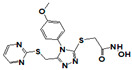	1.3	>17	>17	>17	>17	>17	>17	>17	>17
1596857	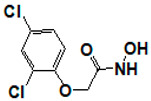	1.18	>17	>17	>17	>17	>17	>17	>17	>17
220670	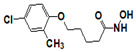	1.12	>17	>17	>17	4.20	17	>17	>17	>17
162799	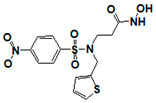	0.564	>17	>17	>17	>17	>17	>17	>17	>17
162808	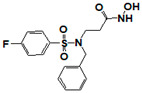	0.446	>17	17	>17	>17	>17	>17	>17	>17

**Table 3:** Selectivity testing of meprin β top HTS hits. All units are IC_50_, µM.

Compound ID	Structure	Meprin α	Meprin β	MMP-2	MMP-3	MMP-8	MMP-9	MMP-10	MMP-14	ADAM17
SR207820	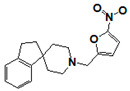	>17	1.5	>17	17	>17	>17	>17	>17	>17
SR412882	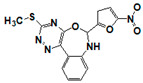	>17	3.5	>17	15	>17	>17	9.7	10.5	17
SR910128	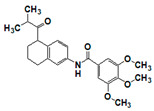	>17	1.0	>17	>17	3.1	4.5	>17	>17	>17
SR910130	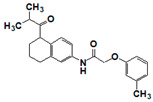	>17	2.0	>17	>17	3.0	9.9	>17	>17	>17
SR910140	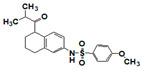	>17	1.6	>17	>17	4.0	10	>17	>17	>17
SR355996	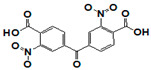	>17	0.97	>17	>17	>17	17	>17	>17	>17

**Table 4:** Comparison of SR162808 and compounds 10d and 10e from **^17^**. All units are IC_50_, µM.

ID	Meprin α	Meprin β	Selectivity Fold
SR162808	0.30	>17	38
10d	0.16	2.95	18
10e	0.40	7.59	19

## Data Availability

All data are available upon request.

## Abbreviations

uHTS	ultrahigh-throughput screening
MMP	matrix metalloprotease
ADAM	a disintegrin and metalloprotease
